# Amivantamab and Mobocertinib in Exon 20 insertions EGFR Mutant Lung Cancer, Challenge To The Current Guidelines

**DOI:** 10.1016/j.tranon.2022.101475

**Published:** 2022-07-01

**Authors:** Timothée Olivier, Vinay Prasad

**Affiliations:** a Department of Oncology, Geneva University Hospital, 4 Gabrielle-Perret-Gentil Street, 1205, Geneva, Switzerland; b Department of Epidemiology and Biostatistics, University of California San Francisco, 550 16th St, 2nd Fl, San Francisco, CA 94158, USA

**Keywords:** EGFR, Exon 20 insertion, Guidelines, Non small cell lung cancer

## Abstract

In 2021, the US Food and Drug Administration (FDA) approved two drugs targeting exon 20 directly: amivantamab and mobocertinib, under the accelerated approval pathway, for adult patients with locally advanced or metastatic non-small cell lung cancer (NSCLC) with epidermal growth factor receptor (EGFR) exon 20 insertion mutations, whose disease has progressed on or after platinum-based chemotherapy. Here we discuss questions regarding the core question of an “unmet need” within the accelerated approval pathway, contending that equipoise remain between the new compounds and previously existing options. Second, the NCCN's guidelines are currently recommending to sequence both drugs, a recommendation that is not based on any data. Last, post-marketing requirements may not shed clarity in the setting of these approvals. Our analysis has implications beyond patients with exon 20 insertion. In an era with growing identification of new and rarer molecular entities, misguided incorporation of new compounds into practice may obstruct trial enrollment in decisive clinical trials.

## Graphical Abstract

**Figure:**
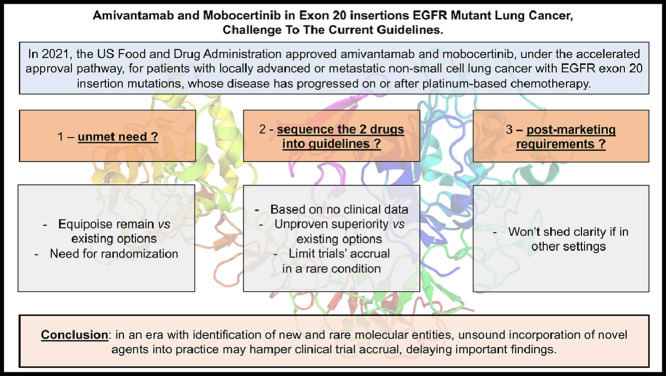

## Manuscript

Treatment options for metastatic EGFR mutant non-small cell lung cancer (NSCLC) have increased during the past two decades. Three generations of EGFR tyrosine kinase inhibitors (TKIs) have been developed targeting more than 90% of EGFR alterations: namely exon 19 deletions and exon 21 mutations. Exon 20 insertion mutations within the EGFR gene are a much rarer genomic event across tumor types, reported in 0.35% of cancers, and 6% of EGFR-mutant lung cancers [1]. First to third generation EGFR TKIs show poor activity in exon 20 insertions.

In 2021, the US Food and Drug Administration (FDA) approved two drugs targeting exon 20 directly: amivantamab and mobocertinib, under the accelerated approval pathway, for “adult patients with locally advanced or metastatic non-small cell lung cancer (NSCLC) with epidermal growth factor receptor (EGFR) exon 20 insertion mutations, as detected by an FDA-approved test, whose disease has progressed on or after platinum-based chemotherapy”. Here we discuss questions regarding (1) the core question of an “unmet need” within the accelerated approval pathway; (2) the NCCN's recommendation to sequence the drugs; (3) and concerns about post-marketing requirements. We contend that overhasty and unsound recommendation may hamper optimal accrual of patients - with a rare condition - into critical clinical trials.

## Debatable qualification of an unmet need

Amivantamab, a bi-specific antibody, targeting the MET-receptor and the EGF-receptor (EGFR), was approved under the accelerated approval pathway on May 21, 2021. The approval was based on data from 81 NSCLC patients within the non-randomized multicohort trial CHRYSALYS (NCT02609776) trial, which showed, in previously treated patients harboring exon 20 insertion mutations, 40 % overall response rate (ORR), 11.1 months median duration of response (DOR), a median progression free survival (PFS) of 8.3 months. The median overall survival (OS) was 22.8 months (14.6 to not reached) [2].

Mobocertinib was the second agent to be approved in the same indication on September 15, 2021. Mobocertinib is an irreversible TKI, inhibiting EGFR activity through covalent and irreversible bond with cysteine 797 within the EGFR protein. The approval was based on data from Study 101 (NCT02716116), an open label multicohort non-randomized trial, which included 114 NSCLC patients with exon 20 insertion mutations, whose disease had progressed on or after platinum-based therapy. Results were a 28% ORR and a median DOR of 17.5 months. Median OS was 24.0 months (14.6 to 28.8). The oral route of administration of mobocertinib may be viewed as an advantage [3].

Among the core principles of the accelerated approval pathway is to qualify an unmet need. The accelerated pathway allows patients to receive the new therapy while waiting for confirmatory data. Current treatment options in the second line setting are single agent chemotherapy, docetaxel in combination with ramucirumab or anti-PD(L1) monotherapy if not received previously, showing response rates from 6 to 23 % and median duration of response from 4 to 17 months. However, these data derive from trials including unselected patients, without specifying outcomes of patients with Exon 20 insertion mutant lung cancer. What are the data for patients with Exon 20 insertion mutations, and how do they compare with those from the 2 approved drugs?

Two retrospective studies from the Flatiron Health database found first-line median OS of 17.0 months in patients with Exon 20 insertion mutation, compared with 9.3 months in unselected patients [4,5]. In real world data from Korea, median OS after diagnosis in patients with Exon 20 insertions was 29.4 months (9.3 to 49.6), response rate in treated patients was 50%, with a 4.2 months PFS [6]. In the relapse or refractory setting, real-world data showed a median OS of 17.1 months (8.3 to 30.0) in Exon 20 insertion mutant lung cancer patients treated by chemotherapy [7]. Regarding immune checkpoint inhibitors direct efficacy in Exon 20 insertion mutant lung cancers, data from retrospective studies suggested similar outcomes as compared to wild-type historical control, and better than classical EGFR mutant patient [8,9]. Taken together, these data suggest (1) better outcome in patients harboring Exon 20 insertions comparted to unselected patients; (2) and sensitivity to chemotherapy.

**Fig. 1:**
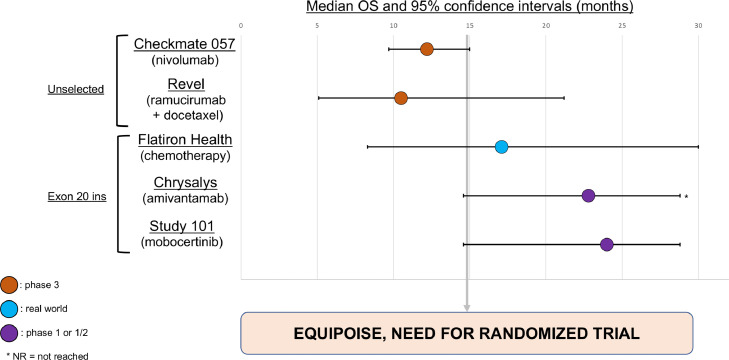
Selected Studies In Second Line Metastatic NSCLC, With Overall Survival (OS).

In other words, outcomes are broadly comparable. We contend that only randomization is capable of adjudication which is preferable between available options or these new agents. The Fig. 1 illustrate the overlapping confidence intervals for the overall survival endpoint of data of existing options in unselected and patients with exon 20 insertions.

## Unproven sequence into guidelines

Second, since version 6.2021 of the NCCN-guidelines (and currently in version 3.2022), both amivantamab and mobocertinib were implemented into treatment algorithm for patients harboring exon 20 insertions mutations. The guidelines specify that patients should shift to the other drug before considering other options. No published or presented data, to date, have assessed the efficacy of amivantamab after mobocertinib, or the other way round.

Beyond the logic of “targeting the target”, this recommendation is not supported by any evidence. We have no data to support that the proposed sequence is superior to existing options. It is possible that oncogenic-driven tumors, like in exon 20 insertions cancers, may benefit from a window between the administration of these drugs. Less selective pressure on the specific exon 20 insertion target could delay or lessen clonal selection and resistance: only trials could define the best sequences of treatments.

Regrettably, in such rare conditions, allowing unproven strategies to be prescribed based on guidelines may limit accrual of patients into meaningful clinical trials.

## Post-marketing requirements

Third, post-marketing requirements of amivantamab are based on the expected results from the PAPILLON trial (NCT04538664), an open phase 3 trial investigating amivantamab in combination with carboplatin-pemetrexed against carboplatin-pemetrexed, whatever the setting (first, second, subsequent line), in NSCLC with exon 20 insertion mutations. Consequently, potential conversion of single agent amivantamab into a regular approval, in a setting where other options previously existed, may rely on a combination trial conducted in another setting. The question of the superiority of single agent amivantamab in relapsing patients over existing options will remained unanswered. Relative to post-marketing requirements of mobocertinib, we don't know in which setting the randomized confirmatory trial will be conducted, as of May 2022.

## Conclusion

Targeting what has hitherto been untargeted is widely and easily celebrated but should not automatically consider an “unmet need”. In this case, alternatives exist with comparable OS, despite apples (all comers) and oranges (exon 20 insertions patients) comparison. The NCCN has drifted beyond evidence to recommend an unproven sequence of two recently approved drugs. Finally, confirmatory trials could not shed clarity. These drugs may be important advances in the treatment of exon 20 mutation diseases; however, clinician will struggle to know how to incorporate the agents based on the scant existing data. Broadly, in an era with growing identification of new and rarer molecular entities, unsound incorporation of new compounds into practice may hamper clinical trial accrual, thus preventing to provide meaningful answers to patients.

## Authors contribution statement

Authors’ contributions: VP contributed to the conception. TO wrote first draft of manuscript and all authors reviewed and revised the manuscript. All authors provided final approval of the manuscript.

## Funding

This project was funded by Arnold Ventures, LLC through a grant paid to the University of California, San Francisco.

## Conflict of interest statements

Vinay Prasad's Disclosures: Research funding: Arnold Ventures; Royalties: Johns Hopkins Press, Medscape; Honoraria: Grand Rounds/lectures from universities, medical centers, non-profits, and professional societies; Consulting: UnitedHealthcare; Speaking fees: Evicore; Other: Plenary Session podcast has Patreon backers. Timothée Olivier have no financial nor non-financial conflicts of interest to report.
